# Pre-operative evaluation and mid-term outcomes of anomalous origin of the left coronary artery from the pulmonary artery based on left ventricular ejection fraction

**DOI:** 10.3389/fcvm.2022.961491

**Published:** 2022-08-09

**Authors:** Shu-Liang Xia, Hui-Kang Tao, Li Ma, Yan-Qing Cui, Ming-Hui Zou, Jian-Ru Li, Feng-xiang Li, Jia Li, Xu Zhang, Xin-Xin Chen

**Affiliations:** ^1^Guangdong Provincial People's Hospital (Guangdong Academy of Medical Sciences), Southern Medical University, Guangdong, China; ^2^Department of Cardiovascular Surgery, Guangzhou Women and Children's Medical Center, Guangzhou Medical University, Guangdong Provincial Clinical Research Center for Child Health, Guangzhou, China; ^3^Department of Pediatric Cardiology, Guangdong Cardiovascular Institute, Guangdong Provincial Key Laboratory of South China Structural Heart Disease, Guangdong, China; ^4^Guangdong Provincial Key Laboratory of Research in Structural Birth Defect Disease, Department of Pediatric Surgery, Guangzhou Women and Children's Medical Center, Guangzhou Medical University, Guangdong Provincial Clinical Research Center for Child Health, Guangzhou, China; ^5^Department of Echocardiogram Room, Guangzhou Women and Children's Medical Center, Guangzhou Medical University, Guangdong Provincial Clinical Research Center for Child Health, Guangzhou, China; ^6^Clinical Physiology Laboratory, Guangzhou Women and Children's Medical Center, Institute of Pediatrics, Guangzhou Medical University, Guangdong Provincial Clinical Research Center for Child Health, Guangzhou, China; ^7^Department of Pediatric Cardiology, Guangzhou Women and Children's Medical Center, Guangzhou Medical University, Guangdong Provincial Clinical Research Center for Child Health, Guangzhou, China

**Keywords:** anomalous left coronary artery originating from the pulmonary artery, coronary artery reimplantation, pre-operative evaluation, effect, infant

## Abstract

**Objective:**

The purpose of this study was to evaluate the prognosis of patients with anomalous left coronary artery originating from pulmonary artery with varying cardiac function after surgical correction.

**Methods:**

This was a single-center retrospective cohort study including 51 patients with anomalous left coronary artery originating from pulmonary artery, all of whom underwent surgery at our center.

**Results:**

All 5 deaths occurred in the pre-operative low cardiac function group (*n* = 39). After corrected by body surface area, parameters such as left coronary artery, right coronary artery, left atrial diameter, left ventricular end-diastolic diameter, left ventricular end-systolic diameter, and main pulmonary artery diameter, were lower in patients in the normal cardiac function group than in the low cardiac function group. The rate of collateral circulation formation was higher in the normal cardiac function group. The proportion of changes of T wave was higher in the low cardiac function group (*P* = 0.005), and the duration of vasoactive drugs (dopamine, milrinone, epinephrine, nitroglycerin.) was longer in the low cardiac function group. Left ventricular end-diastolic diameter, left ventricular end-systolic diameter, main pulmonary artery diameter, and left atrial diameter were smaller than those pre-operatively (*P* < 0.05). Left ventricular ejection fraction was higher than that pre-operatively (*P* = 0.003). The degree of mitral regurgitation in the low cardiac function group was reduced post-operatively (*P* < 0.001).

**Conclusion:**

There was a significant difference between the pre-operative baseline data of the low cardiac function group and the normal cardiac function group. After surgical repair, cardiac function gradually returned to normal in the low cardiac function group. The low cardiac function group required vasoactive drugs for a longer period of time. The left ventricular end-diastolic diameter, left ventricular end-systolic diameter, left atrial diameter, and main pulmonary artery diameter decreased and gradually returned to normal after surgery. The degree of mitral regurgitation in the low cardiac function group was reduced after surgery.

## Introduction

Anomalous origin of the left coronary artery from the pulmonary artery (ALCAPA) is a rare congenital heart disease with an incidence of 1 in 300,000 live births (1). It can cause mitral regurgitation (MR) and left heart dysfunction. If left untreated, the mortality rate in the first year of life is about 90% (2).

ALCAPA can be divided into two types based on whether collateral circulation are abundant or not: infantile and adult types. The infantile type lacks a well-developed collateral circulation from the right coronary artery, leading to early symptoms in infancy. The adult type rarely presents with early symptoms because of a well-developed collateral circulation. However, older patients still have varying degrees of myocardial ischemia, and 80% to 90% of patients are at risk of sudden death (3). In recent years, with advances in echocardiographic and surgical techniques, the mortality rate of ALCAPA has been reduced to a low level (4). Previous studies concluded that low left ventricular ejection fraction (LVEF) is an independent risk factor for death (5–9). However, the clinical characteristics and surgical outcomes of patients with low LVEF are unclear and have not been reported.

In this study, we aimed to investigate the clinical characteristics and mid-term effects of ALCAPA repair, especially in patients with low LVEF, by analyzing the clinical data of children with ALCAPA in our center.

## Patients and methods

This was a retrospective study of consecutive patients who underwent ALCAPA repair at our center from 2011 to 2020. This study was approved by the Ethics Committee, and no individual consent was required.

Overall, 51 patients who were diagnosed with ALCAPA and underwent surgical correction at our hospital were included in this study. Medical records were reviewed, including the following data: baseline details, clinical assessments, surgical management, post-operative outcomes, and clinical assessments at regular follow-up. Clinical assessments included electrocardiogram (ECG), chest radiograph, transthoracic echocardiogram and enhanced cardiac CT scan.

An abnormal Q wave was defined as a duration of ≥0.03 s, depth extending beyond one-quarter of the height of the R wave in the same lead, or depth ≥3 mm in lead I, 2 mm in lead avL, and ST-segment depression referring to depression ≥0.05 mV. Echocardiographic parameters included left atrial diameter (LAD), left ventricular end-systolic diameter (LVESD), left ventricular end-diastolic diameter (LVEDD), main pulmonary artery diameter (MPAD), right ventricular outflow tract diameter (RVOT), and left ventricular ejection fraction (LVEF).

Based on pre-operative LVEF, patients were divided into two groups: low LVEF group (LVEF ≤ 55%) and normal LVEF group (LVEF >55%) (10). The degree of MR on echocardiography was defined as absent, mild, moderate or severe using the ratio of regurgitant jets to the left atrium.

Surgical strategies included coronary artery reimplantation, intrapulmonary baffle repair (Takeuchi procedure), coronary artery bypass grafting, and left coronary artery ligation. Hospital mortality was defined as death before discharge or within 30 days after surgery.

### Statistical analysis

All analyses were performed with IBM SPSS Statistics Version 22 (SPSS, Inc., Armonk, NY, USA). Continuous variables were expressed as mean and standard deviation (SD) or median and interquartile range (IQR). Categorical variables were expressed as frequencies and percentages. Two-tailed *P*-values <0.05 were considered statistically significant.

## Results

### Pre-operative data

The pre-operative data of ALCAPA were summarized in Table 1. Fifty one patients were enrolled and divided into two groups based on pre-operative LVEF: the low LVEF group (LVEF ≤ 55%, *n* = 39) and the normal LVEF group (LVEF >55%, *n* = 12). 43.1% (*n* = 22) of the total patients were male, and 64.7% (*n* = 33) were younger than 1 year of age. The median age at surgery was 0.4 years in the low LVEF group and 3 years in the normal LVEF group (*P* = 0.011).

**Table 1:** Pre-operative data of patients.

	**Low LVEF group (*n* = 39)**	**Normal LVEF group (*n* = 12)**	***p*-value**
Male (*n*)	17	5	0.906
Age (years)	0.4 (0.2, 0.8)	3 (0.5, 8)	0.011
Body weight (kg)	5 (4.5, 8)	15.7 (6.02, 22.75)	0.016
Cardiothoracic ratio	0.667 ± 0.0502	0.594 ± 0.046	<0.001
ECG
Abnormal Q wave	5	1	0.999
Changes of T wave	30	4	0.005
Pre-operative LVEF (%)	27 (19, 37)	62 (58, 69)	<0.001
LAD/BSA (mm/m^2^)	65.58 ± 17.29	41.81 ± 19.32	<0.001
MPAD/BSA (mm/m^2^)	43.66 ± 6.19	29.68 ± 12.11	<0.001
RVOT/BSA (mm/m^2^)	32.84 ± 8.33	29.56 ± 16.95	0.367
LVEDD/BSA (mm/m^2^)	132.25 ± 28.00	69.46 ± 28.74	<0.001
LVESD/BSA (mm/m^2^)	113.59 ± 27.78	45.68 ± 20.00	<0.001
MR degree (*n*)			0.561
None	5	3	
Mild	9	4	
Moderate	16	3	
Severe	9	2	
RCA/BSA (mm/m^2^)	8.23 ± 1.91	7.42 ± 2.76	0.005
LCA/BSA (mm/m^2^)	6.69 ± 1.72	6.09 ± 2.27	0.005
Collateral circulation (*n*)	8	6	0.005

*ECG, electrocardiogram; LVEF, left ventricular ejection fraction; LAD, Left atrial diameter; MPAD, main pulmonary artery diameter; RVOT, right ventricular outflow tract diameter; LVEDD, LV end-diastolic diameter; LVESD, LV end-systolic diameter; RCA, right coronary artery; LCA, left coronary artery; BSA, body surface area; MR, mitral regurgitation*.

In the low LVEF group, the major complaints were hyperhidrosis in 14 patients (35%), feeding difficulties in 9 (23%), syncope in 2 (5%), and severe heart failure in 1 (2.5%). The initial diagnoses were endocardial fibroelastosis (EFE) in 6 (15%), dilated cardiomyopathy (DCM) in 10 (25%), coronary artery fistula in 1 (2.5%), and ALCAPA in 21 (53%). In the normal LVEF group, 2 patients (16%) presented as hyperhidrosis, and 9 (75%) as incidental finding of cardiac murmur.

The ECGs showed abnormal Q waves in leads I, aVL, V5, and V6 in 6 patients (low LVEF group vs. normal LVEF group: 5 vs. 1, *P* = 0.999) and T wave changes in 34 patients (low LVEF group vs. normal LVEF group: 30 vs. 4, *P* = 0.0051). The baseline date of echocardiogram and diameter of coronary artery were summarized in Supplementary Table 1. To better compare the data between the two groups, we normalized the data by body surface area (BSA). The diameter of RVOT/BSA was not significantly different between the groups (32.84 ± 8.33 vs. 29.56 ± 16.95, *p* = 0.367). In contrast, the diameter of LCA/BSA, RCA/BSA, LAD/BSA, LVEDD/BSA, LVESD/BSA and MPAD/BSA were significantly higher in the low LVEF group than in the normal LVEF group (LCA/BSA 6.69 ± 1.72 vs. 6.09 ± 2.27, *p* = 0.005; RCA/BSA 8.23 ± 1.91 vs 7.42 ± 2.76, *p* = 0.005; LAD/BSA 65.58 ± 17.29 vs. 41.81 ± 19. 32, *p* < 0.001; LVEDD/BSA 132.25 ± 28 vs. 69.46 ± 28.74, *p* < 0.001; LVESD/BSA 113.59 ± 27.78 vs. 45.68 ± 20, *p* < 0.001; MPAD/BSA 43.66 ± 6.19 vs. 29.68 ± 12.11 *p* < 0.001). There was no significant difference in MR percentage between the groups (*p* = 0.561). The normal LVEF group had a greater percentage of collateral circulation (*p* = 0.005). The cardiothoracic ratio was significantly higher in the low LVEF group compared to the normal LVEF group (0.66 ± 0.050 vs. 0.59 ± 0.046, *p* < 0.001).

### Cardiac surgery

All patients underwent coronary artery reimplantation except one patient in the normal LVEF group who underwent left coronary artery ligation because of a well-developed collateral circulation. There was no significant difference in the proportion of patients who underwent simultaneous mitral valvuloplasty in the two groups (*P* = 0.815). Cardiopulmonary bypass (CPB) time (184.38 ± 85.99 vs. 131.75 ± 55.98 min, *P* = 0.053) and aortic cross clamping time (72.64 ± 26.27 vs. 71.92 ± 35.71 min, *P* = 0.94) were also not significant between the two groups.

Table 2 summarizes the outcomes of patients with ALCAPA repair. All patients were treated in the CICU post-operatively. Mechanical ventilation time (140.80 ± 150.71 vs. 34.56 ± 42.77 min, *p* = 0.057) was not significant between the two groups. However, duration of milrinone (166.09 ± 102.78 vs. 64.4 ± 43.78 min, *p* = 0.004), dopamine (195.02 ± 140.7 vs. 62.7 ± 42.5 min, *p* = 0.006), nitroglycerin (130.75 ± 93.56 vs. 55.43 ± 28.92 min, *p* = 0.045) and epinephrine (138.13 ± 106.56 vs. 43.83 ± 48.18 min, *p* = 0.043) were longer in the low LVEF group than in the normal LVEF group.

**Table 2:** Patients' characteristics and outcomes.

	**Low LVEF group (*n* = 39)**	**Normal LVEF group (*n* = 12)**	***p*-value**
Operation time (min)	232.5 (183.75, 318.75)	240 (180, 255)	0.04
CPB time (min)	184.38 ± 85.99	131.75 ± 55.98	0.053
Aortic cross-clamp time (min)	72.64 ± 26.27	71.92 ± 35.71	0.94
MV repair (*n*)	21	6	0.815
Drugs use time (min)
Milrinone	166.09 ± 102.78	64.4 ± 43.78	0.004
Dopamine	195.02 ± 140.7	62.7 ± 42.5	0.006
Nitroglycerin	130.75 ± 93.56	55.43 ± 28.92	0.045
Epinephrine	138.13 ± 106.56	43.83 ± 48.18	0.043
Mechanical ventilation time (min)	140.80 ± 150.71	34.56 ± 42.77	0.057
Complications (*n*)
Chylothorax	1	0	
Delayed sternal closure	7	0	
Arrhythmia	6	0	
LVEF at discharge (%)	36.87 ± 16.28	63.35 ± 9.7	<0.001
Cardiothoracic ratio at discharge	0.64 ± 0.05	0.58 ± 0.05	0.002
Hospital mortality (*n*)	5	0	
Mid-term mortality (*n*)	1	0	
Mid-term MV repair (*n*)	1	0	

*LVEF, left ventricular ejection fraction; CBP, cardiopulmonary by-pass*.

**Figure 1:**
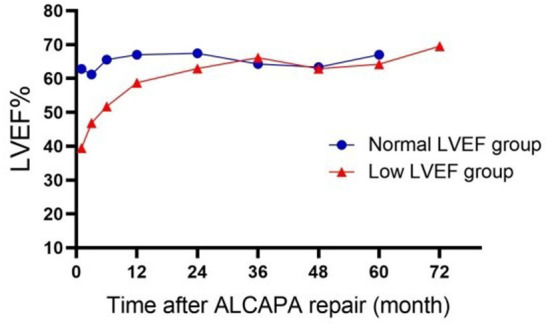
Dynamic change of LVEF after ALCAPA repair. LVEF, left ventricular ejection fraction.

At discharge, there were no significant changes in the diameter of RVOT, cardiothoracic ratio, and the proportion of abnormal Q and T waves compared to pre-operative counterparts (RVOT, 11.48 ± 3.63 vs. 11.26 ± 3.27, *p* = 0.684; cardiothoracic ratio, 0.627 ± 0.054 vs. 0.616 ± 0.06, *p* = 0.253; abnormal Q waves, *p* = 0.269; T wave changes, *p* = 0.308). There was a decrease in the diameters of LAD, MPAD, LVEDD and LVESD at discharge (Table 3, LAD 20.96 ± 5.81 vs. 18.3 ± 4.34, *p* = 0.001; MPAD 14.30 ± 3.35 vs. 13.54 ± 3.07, *p* = 0.004; LVEDD 40.46 ± 10.5 vs. 32.47 ± 10.51, *p* < 0.001; LVESD 32.68 ± 10.87 vs. 26.3 ± 11.12, *p* = 0.001). LVEF was improved at discharge either in all patients [(37.86 ± 19.3)% vs. (43.34 ± 19.06)%, *p* = 0.003], or in the low LVEF group [(28.48 ± 13.13)% vs. (36.87 ± 16.28)%, *p* < 0.001] (Figure 1). In the low LVEF group, the percentage of moderate to severe MR decreased significantly from 64.1% to 26.4% at discharge (*p* < 0.001, Table 4). In the normal LVEF group, the LVEF [(65.58 ± 6.08)% vs. (63.35 ± 9.7)%, *p* = 0.863] and the percentage of moderate to severe MR at discharge were not significant (*p* = 0.179).

**Table 3:** Comparison between pre- and post-operative data.

	**Pre-operative (*n* = 46)**	**Post-operative (*n* = 46)**	***p-value***
LAD (mm)	20.96 ± 5.81	18.30 ± 4.34	0.001
RVOT (mm)	11.48 ± 3.63	11.26 ± 3.27	0.684
MPAD (mm)	14.30 ± 3.35	13.54 ± 3.07	0.004
LVEDD (mm)	40.46 ± 10.5	32.47 ± 10.51	<0.001
LVESD (mm)	32.68 ± 10.87	26.30 ± 11.12	0.001
LVEF (%)	37.86 ± 19.3	43.34 ± 19.06	0.003
ECG (*n*)
Abnormal Q wave	6	2	0.269
Changes of T wave	34	29	0.308
Cardiothoracic ratio	0.627 ± 0.054	0.616 ± 0.06	0.253

*LVEF, left ventricular ejection fraction; LAD, Left atrial diameter; MPAD, main pulmonary artery diameter; RVOT, right ventricular outflow tract diameter; LVEDD, LV end-diastolic diameter; LVESD, LV end-systolic diameter*.

**Table 4:** Comparison between pre- and post-operative MR degree.

	**Pre-operative**	**Post-operative**	***p*-value**
MR degree (Low LVEF group, *n*)			<0.001
None	5	5	
Mild	9	20	
Moderate	16	8	
Severe	9	1	
MR degree (Normal LVEF group, *n*)			0.179
None	3	1	
Mild	4	5	
Moderate	3	2	
Severe	2	0	

### Mortality and post-operative complications

Hospital mortality was 9.8% (5 of 51). No patient died in the normal LVEF group. Three patients died of heart failure, and one of them received repetitive mitral valvuloplasty due to severe MR. One died of malignant arrhythmia and the other died of respiratory failure due to a severe pulmonary infection. All of them were post-operative death.

No complications occurred in the normal LVEF group. No patient required extracorporeal membrane oxygenation (ECMO) support post-operatively. Delayed sternal closure was present in 7 (13.7%) patients due to unstable perioperative hemodynamics. Chylothorax was seen in one patient (1.9%) and improved with conservative treatment. Arrhythmias were present in 6 (11.8%) patients, two of whom received temporary cardiac pacing.

### Follow-up

The median duration of follow-up was 3 (2.6–3.3) years. The longest follow-up was 9 years. There was no late mortality in the normal LVEF group, while one patient died of accidentally fall 1 year after the procedure in the low LVEF group. In the low LVEF group, one patient underwent mitral valvuloplasty for severe MR and infective endocarditis 6 years after LCA implantation. LVEF in the low LVEF group returned to normal within a median time of 12 months (Figure 1). At the last follow-up, nine patients in the low LVEF group had moderate or severe MR and four patients in the normal LVEF group (*p* = 0.4743) with normal LV function.

## Discussion

Misdiagnosis of ALCAPA patients of different ages frequently occurs due to lack of specificity in clinical presentation, ranging from asymptomatic to heart failure and sudden death. In our study, 27 patients (52%) had false initial diagnoses due to lack of specificity of clinical presentation. In the neonatal period, pulmonary vascular resistance is high and abnormal LCA is supplied by PA. Despite relatively low oxygen saturation, the effective perfusion pressure was high enough to exclude significant myocardial ischemia.

At 1 month after birth, pulmonary vascular resistance falls to normal level, so that LCA is supplied by coronary artery bypass of the RCA. Myocardial ischemia and fibrosis may develop, leading to LV dilation and impaired cardiac function. Dyspnea, feeding intolerance, hyperhidrosis and inability to grow are the most common manifestations. Chest radiographs always show an enlarged heart. Based on pre-operative ECG data, most patients have distinct ECG features: abnormal Q-wave and T-wave inversions in leads I, aVL and V4-V6, especially in aVL, which can be helpful in the differential diagnosis (11). Echocardiography of ALCAPA shows no obvious vascular structures at the origin of the LCA and abnormal pulmonary artery blood flow. Some patients have concomitant LV systolic dysfunction, MR and LV enlargement. In our study, we found that patients with ALCAPA had varying degrees of MR and enlarged LV, especially those with low LVEF. Several studies have suggested that echocardiography can easily be misdiagnosed as dilated cardiomyopathy or endocardial fibroelastosis (12, 13). Typical ECG changes can provide valuable clues to detect the presence of ALCAPA in suspicious patients. If ALCAPA is highly suspicious or confirmed after echocardiography, it should be confirmed by CT scan or angiography before cardiac surgery. In our study, the results of CT scans showed that the origin of the abnormal coronary artery was found in all patients. Therefore, cardiac enhanced CT scans are recommended for patients suspected of having ALCAPA.

In the current study, patients were divided into two groups based on pre-operative LVEF. The mean age of the low LVEF group was 0.4 years, most of which were infants, while the mean age of the normal LVEF group was 3 years, most of which were elderly patients. The normal LVEF group had more collateral circulation than the low LVEF group. This suggests that the cardiac function of ALCAPA patients is related to the collateral circulation. Low LVEF has been reported to be a risk factor for death in ALCAPA (5–8). In-hospital mortality after ALCAPA repair has been reported to range from 0 to 16% (14–16). In our study, the hospital mortality rate after surgery was 9.8% (5/51), which is comparable to the results of other centers. The five patients who died belonged to the low LVEF group and the main cause of death was heart failure or respiratory failure, suggesting that low LVEF is a risk factor for death 30 days after surgery. Over time, the LVEF improved and was within the normal range in most of our patients. An improvement in LVEF was observed at 1 year post-operatively. Some studies suggest that this may be due to the restoration of blood flow to previously hibernating myocardium (8, 17).

The primary treatment for ALCAPA is surgery, including coronary artery bypass grafting, coronary artery reimplantation, Takeuchi procedure and coronary artery ligation. The aim of surgery is to reconstruct the normal coronary artery system. With the accumulation of experience in coronary artery grafting during transposition of the great arteries, direct coronary artery reimplantation has become the first choice for ALCAPA (18, 19). In the present study, all patients underwent coronary artery reimplantation except one in the normal LVEF group who underwent left coronary artery ligation due to extensive collaterals around the pulmonary sinus. It remains controversial for treating MR at the time of ALCAPA repair (20). The severity of MR has been reported as a risk factor for mortality and reoperation rates (5, 7). Most studies have concluded that treatment of MR is effective in promoting early recovery of cardiac function after ALCAPA repair (8, 9), although controversies still exist. Approaches to mitral valvuloplasty include intermittent valvuloplasty and fibrous papillary muscle stripping. In our study, pre-operative echocardiography showed no difference in MR ratios between the two groups of patients. We performed simultaneous MV repair in 27 patients. There was a decrease in the ratio of moderate and severe MR at discharge compared to the pre-operative period. One patient died of heart failure due to worsening MR. During follow-up, 13 patients still had moderate or severe MR but LV function had been normalized, and one patient underwent a second mitral valvuloplasty for heart failure due to severe MR. These results suggested that not all MR improve gradually by restoring LV function.

Patients with ALCAPA have inadequate post-operative cardiac function due to the long duration of aortic clamping and ischemia-reperfusion during surgery. Our results showed that the perioperative duration of vasoactive drugs was longer in the low LVEF group than in the normal LVEF group, suggesting that the maintenance of LV function after ALCAPA repair depends mainly on the use of inotropes, diuretics and vasodilators. Pharmacological treatment of chronic heart failure should be maintained after surgery until LV function and size return to normal range. This is an important factor in improving long-term prognosis, especially in patients with low LVEF (21). Long-term survival rates for patients with ALCAPA are 86–100% (7, 22). During the follow-up of this study, one patient in the low LVEF group died from accidentally fall, while the long-term survival rate in the normal LVEF group was 100%.

## Conclusion

There was a significant difference between the pre-operative baseline data of the low cardiac function group and the normal cardiac function group. After surgical repair, cardiac function gradually returned to normal in the low cardiac function group. The low cardiac function group required vasoactive drugs for a longer period of time. The LVEDD, LVEDS, LAD, and MPAD decreased and gradually returned to normal after surgery. The degree of MR in the low cardiac function group was reduced after surgery. Low LVEF may be a risk factor for death 30 days after surgery.

## Limitations

Our study has limitations. First, the study design was a retrospective cohort series and the number of patients was small. Second, different surgical management and ALCAPA repair techniques were not compared. Further studies with longer follow-up and larger case numbers will be performed in the future.

## Data availability statement

The original contributions presented in the study are included in the article/Supplementary material, further inquiries can be directed to the corresponding author/s.

## Ethics statement

The studies involving human participants were reviewed and approved by Guangzhou Women and Children's Medical Center. Written informed consent to participate in this study was provided by the participants' legal guardian/next of kin. Written informed consent was obtained from the individual(s), and minor(s)' legal guardian/next of kin, for the publication of any potentially identifiable images or data included in this article.

## Author contributions

S-LX: conceptualization, data curation, investigation, and writing—review and editing. H-KT: conceptualization, data curation, and writing—review and editing. LM: formal analysis and investigation. Y-QC: data curation and formal analysis. J-RL: ultrasound diagnosis and evaluation. F-xL: data curation and investigation. JL: modification. XZ: data curation and modification. X-XC: conceptualization, investigation, and supervision. All authors contributed to the article and approved the submitted version.

## Funding

This study was supported by the Science and Technology Planning Project of Guangzhou (Grant No. 202201020646), Young Scientists Fund of National Natural Science Foundation of China (Grant No. 81900222), Ph.D. Start-up Fund of Guangzhou Women and Children's Medical Center (Grant No. 1600010), and Guangzhou Health science and Technology Project (Grant No. 20211A010026).

## Conflict of interest

The authors declare that the research was conducted in the absence of any commercial or financial relationships that could be construed as a potential conflict of interest.

## Publisher's note

All claims expressed in this article are solely those of the authors and do not necessarily represent those of their affiliated organizations, or those of the publisher, the editors and the reviewers. Any product that may be evaluated in this article, or claim that may be made by its manufacturer, is not guaranteed or endorsed by the publisher.

## Abbreviations

ALCAPA: Anomalous origin of the left coronary artery from the pulmonary artery
MR: Mitral regurgitation
LVEF: Left ventricular ejection fraction
ECG: Electrocardiogram
LAD: Left atrial diameter
LVESD: Left ventricular end-systolic diameter
LVEDD: Left ventricular end-diastolic diameter
MPAD: Main pulmonary artery diameter
RVOT: Right ventricular outflow tract diameter
EFE: Endocardial fibroelastosis
DCM: Dilated cardiomyopathy
BSA: Body surface area
CPB: Cardiopulmonary bypass
ECMO: Extracorporeal membrane oxygenation
RCA: Right coronary artery
LCA: Left coronary artery.

## References

[1] Cheng TO. Anomalous pulmonary origin of the left coronary artery. Circulation. (2002) 105:E63–3. 10.1161/circ.105.10.e63 11889027

[2] Williams IA Gersony WM Hellenbrand WE. Anomalous right coronary artery arising from the pulmonary artery: a report of 7 cases and a review of the literature. Am Heart J. (2006) 152:1004.e9–17. 10.1016/j.ahj.2006.07.023 17070181

[3] Berdjis F Takahashi M Wells WJ Stiles QR Lindesmith GG. Anomalous left coronary artery from the pulmonary artery. Significance of intercoronary collaterals. J Thorac Cardiovasc Surg. (1994) 108:17–20. 10.1016/S0022-5223(94)70212-8 8028363

[4] Furuta A Matsumura G Shinkawa T Niinami H. Long-term surgical results of anomalous origin of the left coronary artery from the pulmonary artery repair in infants and older patients. J Card Surg. (2021) 36:821–7. 10.1111/jocs.15285 33522620

[5] Cabrera AG Chen DW Pignatelli RH Khan MS Jeewa A Mery CM. Outcomes of anomalous left coronary artery from pulmonary artery repair: beyond normal function. Ann Thorac Surg. (2015) 99:1342–7. 10.1016/j.athoracsur.2014.12.035 25725925

[6] Weigand J Marshall CD Bacha EA Chen JM Richmond ME. Repair of anomalous left coronary artery from the pulmonary artery in the modern era: pre-operative predictors of immediate post-operative outcomes and long term cardiac follow-up. Pediatr Cardiol. (2015) 36:489–97. 10.1007/s00246-014-1038-8 25301273

[7] Naimo PS Fricke TA d'Udekem Y Cochrane AD Bullock A Robertson T. Surgical intervention for anomalous origin of left coronary artery from the pulmonary artery in children: a long-term follow-up. Ann Thorac Surg. (2016) 101:1842–8. 10.1016/j.athoracsur.2015.11.020 26897320

[8] Neumann A Sarikouch S Bobylev D Meschenmoser L Breymann T Westhoff-Bleck M. Long-term results after repair of anomalous origin of left coronary artery from the pulmonary artery: Takeuchi repair versus coronary transfer. Eur J Cardiothorac Surg. (2017) 51:308–15. 10.1093/ejcts/ezw268 28186291

[9] Lange R Vogt M Hörer J Cleuziou J Menzel A Holper K. Long-term results of repair of anomalous origin of the left coronary artery from the pulmonary artery. Ann Thorac Surg. (2007) 83:1463–71. 10.1016/j.athoracsur.2006.11.005 17383358

[10] Nadruz W Jr West E Santos M Skali H Groarke JD Forman DE. Heart failure and midrange ejection fraction: implications of recovered ejection fraction for exercise tolerance and outcomes. Circ Heart Fail. (2016) 9:e002826. 10.1161/CIRCHEARTFAILURE.115.002826 27009553 PMC4807736

[11] Mesurolle B Qanadli SD Merad M Mignon F Lacombe P Dubourg O. Anomalous origin of the left coronary artery arising from the pulmonary trunk: report of an adult case with long-term follow-up after surgery. Eur Radiol. (1999) 9:1570–3. 10.1007/s003300050885 10525866

[12] Chang RR Allada V. Electrocardiographic and echocardiographic features that distinguish anomalous origin of the left coronary artery from pulmonary artery from idiopathic dilated cardiomyopathy. Pediatr Cardiol. (2001) 22:3–10. 10.1007/s002460010142 11123118

[13] Yuan XC Hu J Zeng X Zhou AY Chen L. Echocardiographic diagnosis of anomalous origin of the left coronary artery from the pulmonary artery. Medicine. (2019) 98:e18046. 10.1097/MD.0000000000018046 31764828 PMC6882605

[14] Kazmierczak PA Ostrowska K Dryzek P Moll JA Moll JJ. Repair of anomalous origin of the left coronary artery from the pulmonary artery in infants. Interact Cardiovasc Thorac Surg. (2013) 16:797–801. 10.1093/icvts/ivt061 23442939 PMC3653475

[15] Muzaffar T Ahmad Ganie F Gpoal Swamy S Wani NU. The surgical outcome of anomalous origin of the left coronary artery from the pulmonary artery. Int Cardiovasc Res J. (2014) 8:57–60. Available online at: https://brieflands.com/articles/ircrj-12030.html 24936482 PMC4058485

[16] Michielon G Di Carlo D Brancaccio G Guccione P Mazzera E Toscano A. Anomalous coronary artery origin from the pulmonary artery: correlation between surgical timing and left ventricular function recovery. Ann Thorac Surg. (2003) 76:581–8. Discussion 588. 10.1016/S0003-4975(03)00344-8 12902108

[17] Kudumula V Mehta C Stumper O Desai T Chikermane A Miller P. Twenty-year outcome of anomalous origin of left coronary artery from pulmonary artery: management of mitral regurgitation. Ann Thorac Surg. (2014) 97:938–44. 10.1016/j.athoracsur.2013.11.042 24480257

[18] Lambert V Touchot A Losay J Piot JD Henglein D Serraf A. Midterm results after surgical repair of the anomalous origin of the coronary artery. Circulation. (1996) 94(9 Suppl.):II38–43. 8901717

[19] Ramírez S Curi-Curi PJ Calderón-Colmenero J García J Britton C Erdmenger J. Outcomes of coronary reimplantation for correction of anomalous origin of left coronary artery from pulmonary artery. Rev Esp Cardiol. (2011) 64:681–7. 10.1016/j.rec.2011.04.014 21715077

[20] Karimi M Kirshbom PM. Anomalous origins of coronary arteries from the pulmonary artery: a comprehensive review of literature and surgical options. World J Pediatr Congenit Heart Surg. (2015) 6:526–40. 10.1177/2150135115596584 26467866

[21] Chiu HH Wang JK Chen CA Chiu SN Lin MT Lue HC. Resolution of pathologic Q wave, left ventricular dysfunction and mitral regurgitation after dual coronary repair of the anomalous origin of the left coronary artery from the pulmonary artery. Eur J Pediatr. (2008) 167:1277–82. 10.1007/s00431-008-0667-4 18317804

[22] El-Hamamsy I Ibrahim M Yacoub MH. 30-year outcome of anatomic correction of anomalous origin of the left coronary artery from the pulmonary artery. J Am Coll Cardiol. (2011) 57:1493. 10.1016/j.jacc.2010.06.064 21435520

