# Corrigendum: Pre-operative evaluation and mid-term outcomes of anomalous origin of the left coronary artery from the pulmonary artery based on left ventricular ejection fraction

**DOI:** 10.3389/fcvm.2024.1408155

**Published:** 2024-06-05

**Authors:** Shu-Liang Xia, Hui-Kang Tao, Li Ma, Yan-Qing Cui, Ming-Hui Zou, Jian-Ru Li, Feng-xiang Li, Jia Li, Xu Zhang, Xin-Xin Chen

**Affiliations:** ^1^ Guangdong Provincial People’s Hospital (Guangdong Academy of Medical Sciences), Southern Medical University, Guangdong, China; ^2^ Department of Cardiovascular Surgery, Guangzhou Women and Children’s Medical Center, Guangzhou Medical University, Guangdong Provincial Clinical Research Center for Child Health, Guangzhou, China; ^3^ Department of Pediatric Cardiology, Guangdong Cardiovascular Institute, Guangdong Provincial Key Laboratory of South China Structural Heart Disease, Guangdong, China; ^4^ Guangdong Provincial Key Laboratory of Research in Structural Birth Defect Disease, Department of Pediatric Surgery, Guangzhou Women and Children’s Medical Center, Guangzhou Medical University, Guangdong Provincial Clinical Research Center for Child Health, Guangzhou, China; ^5^ Department of Echocardiogram Room, Guangzhou Women and Children’s Medical Center, Guangzhou Medical University, Guangdong Provincial Clinical Research Center for Child Health, Guangzhou, China; ^6^ Clinical Physiology Laboratory, Guangzhou Women and Children’s Medical Center, Institute of Pediatrics, Guangzhou Medical University, Guangdong Provincial Clinical Research Center for Child Health, Guangzhou, China; ^7^ Department of Pediatric Cardiology, Guangzhou Women and Children’s Medical Center, Guangzhou Medical University, Guangdong Provincial Clinical Research Center for Child Health, Guangzhou, China

**Keywords:** anomalous left coronary artery originating from the pulmonary artery, coronary artery reimplantation, pre-operative evaluation, effect, infant

A Corrigendum on Pre-operative evaluation and mid-term outcomes of anomalous origin of the left coronary artery from the pulmonary artery based on left ventricular ejection fraction By Xia S-L, Tao H-K, Ma L, Cui Y-Q, Zou M-H, Li J-R, Li F-x, Li J, Zhang X and Chen X-X (2022). Front. Cardiovasc. Med. 9:961491. doi: 10.3389/fcvm.2022.961491

In the published article, there was an error regarding the affiliations for Shu-Liang Xia. As well as having affiliation(s) 2 and 4, they should also have “Guangdong Provincial People's Hospital (Guangdong Academy of Medical Sciences), Southern Medical University, Guangdong, China” and “Department of Pediatric Cardiology, Guangdong Cardiovascular Institute, Guangdong Provincial Key Laboratory of South China Structural Heart Disease, Guangdong, China”.

In the published article, the acknowledgements section was missing. The **Acknowledgements** statement is below:

The authors would like to thank S-LX's M.D. graduate supervisor, Prof. Zhiwei Zhang, from Guangdong Provincial People's Hospital, Southern Medical University, China, for his help in conceptualization, data compilation, investigation, guidance, writing-reviewing, and editing.

The authors apologize for these errors and state that this does not change the scientific conclusions of the article in any way. The original article has been updated.

