# Supplementary material for: Pre-operative evaluation and mid-term outcomes of anomalous origin of the left coronary artery from the pulmonary artery based on left ventricular ejection fraction
Source: Front Cardiovasc Med. 2022 Aug 9;9:961491. doi: 10.3389/fcvm.2022.961491 (PMC9395579; doi:10.3389/fcvm.2022.961491)
Supplement: Supplementary file 1 [file Data_Sheet_1.pdf]

Supplemental table1 Pre-operative baseline data of patients

	**Low LVEF group(n=39)**	**Normal LVEF group(n=12)**	***p* Value**
LAD(mm)	20.28±5.11	22.76±6.81	0.184
MPAD(mm)	13.72±3.01	16.17±3.56	0.022
RVOT(mm)	10.21±2.57	15.25±3.22	<0.001
LVEDD(mm)	41.05±9.81	38.61±10.87	0.476
LVESD(mm)	35.31±10.02	25.33±7.83	0.003
RCA(mm)	2.59±0.82	4.34±1.49	<0.001
LCA(mm)	2.06±0.55	3.6±1.33	<0.001

Abbreviations: LAD, Left atrial diameter; MPAD, main pulmonary artery diameter; RVOT, right ventricular outflow tract diameter; LVEDD, LV end-diastolic diameter; LVESD, LV end-systolic diameter; RCA, right coronary artery; LCA, left coronary artery.
